# Soluble ST2 Associates with Diabetes but Not Established Cardiovascular Risk Factors: A New Inflammatory Pathway of Relevance to Diabetes?

**DOI:** 10.1371/journal.pone.0047830

**Published:** 2012-10-24

**Authors:** Ashley M. Miller, David Purves, Alex McConnachie, Darren L. Asquith, G. David Batty, Harry Burns, Jonathan Cavanagh, Ian Ford, Jennifer S. McLean, Chris J. Packard, Paul G. Shiels, Helen Turner, Yoga N. Velupillai, Kevin A. Deans, Paul Welsh, Iain B. McInnes, Naveed Sattar

**Affiliations:** 1 Institute of Cardiovascular & Medical Sciences, College of Medical, Veterinary & Life Sciences, University of Glasgow, Glasgow, United Kingdom; 2 Robertson Centre for Biostatistics, University of Glasgow, Glasgow, United Kingdom; 3 Institute of Infection, Immunity and Inflammation, College of Medical, Veterinary & Life Sciences, University of Glasgow, Glasgow, United Kingdom; 4 Department of Epidemiology & Public Health, University College London, London, United Kingdom; 5 Scottish Government, Edinburgh, United Kingdom; 6 Institute of Mental Health and Wellbeing, College of Medical, Veterinary and Life Sciences, Glasgow, United Kingdom; 7 Centre for Population Health, Glasgow, United Kingdom; 8 Glasgow Clinical Research Facility, Western Infirmary, Glasgow, United Kingdom; 9 Institute of Cancer Sciences, College of Medical, Veterinary and Life Sciences, University of Glasgow, Glasgow, United Kingdom; 10 Department of Clinical Biochemistry, Aberdeen Royal Infirmary, Aberdeen, United Kingdom; 11 Graduate Entry Medical School (GEMS), University of Limerick, Limerick, Ireland; German Diabetes Center, Leibniz Center for Diabetes Research at Heinrich Heine University Duesseldorf, Germany

## Abstract

Preliminary data mostly from animal models suggest the sST2/IL-33 pathway may have causal relevance for vascular disease and diabetes and thus point to a potential novel inflammatory link to cardiometabolic disease. However, the characterisation of sST2 levels in terms of metabolic or vascular risk in man is completely lacking. We sought to address this gap via a comprehensive analysis of risk factor and vascular correlates of sST2 in a cross-sectional study (pSoBid). We measured sST2 in plasma in 639 subjects and comprehensively related it to cardiovascular and diabetes risk factors and imaged atherosclerosis measures. Circulating sST2 levels increased with age, were lower in women and in highest earners. After adjusting for age and gender, sST2 levels associated strongly with markers of diabetes, including triglycerides [effect estimate (EE) per 1 standard deviation increase in sST2∶1.05 [95%CI 1.01,1.10]), liver function (alanine aminotransaminase [ALT] and γ-glutamyl transferase [GGT]: EE 1.05 [1.01,1.09] and 1.13 [1.07,1.19] respectively), glucose (1.02 [1.00,1.03]) and sICAM-1 (1.05 [1.02,1.07]). However, sST2 levels were not related to smoking, cholesterol, blood pressure, or atheroma (carotid intima media thickness, plaque presence). These results suggest that sST2 levels, in individuals largely without vascular disease, are related principally to markers associated with diabetes and ectopic fat and add support for a role of sST2 in diabetes. Further mechanistic studies determining how sST2 is linked to diabetes pathways may offer new insights into the inflammatory paradigm for type 2 diabetes.

## Introduction

Type 2 diabetes (T2D) is now a global epidemic associated with increased risk of cardiovascular disease (CVD), including coronary heart disease and stroke and selected cancers [1], [2]. The prediction of diabetic risk in the general population continues to be a major challenge. The identification of additional novel biomarkers will serve to elucidate underlying pathophysiological mechanisms, and may allow prediction of downstream complications or the identification of sub-groups of patients exhibiting distinct therapeutic responses [3]. A large body of evidence points towards the involvement of subclinical, low-grade inflammation and activation of the innate immune system, including increased systemic levels of acute phase proteins, cytokines and adhesion molecules in patients with T2D [4], [5].

ST2 (also known as IL-1RL1, OMIM *601203) is a member of the Toll-like/Interleukin (IL)-1 cytokine receptor super-family [6]. The ST2 gene encodes 2 protein isoforms formed by alternative mRNA splicing. ST2L is a trans-membrane receptor comprising 3 linked extracellular immunoglobulin domains, a single trans-membrane domain, and an intracellular Toll-interleukin-1 receptor (TIR) domain homologous to toll-like receptors, which signal through IL-1RAcP. Soluble ST2 (sST2) is a secreted form lacking the trans-membrane and intracellular domains, and is thought to function as a decoy receptor. The ligand for ST2 is IL-33 (also known as IL-1F11), a recently identified member of the IL-1 cytokine family that exhibits a broad range of immune regulatory functions [7]. IL-33 appears to exert protective cardiovascular effects in mice, including reduced atherosclerotic burden in ApoE^−/−^ mice, reduced adiposity with reduced fasting glucose and improved glucose/insulin tolerance in *ob/ob* mice, and reduced cardiac hypertrophy and fibrosis in a murine model of pressure overload [8], [9], [10]. Furthermore, ApoE^−/−^ mice treated with sST2 developed significantly larger atherosclerotic plaques in the aortic sinus compared to control mice, and sST2 reversed the anti-hypertrophic effects of IL-33 on cultured cardiomyocytes [10] . Thus, the soluble form of ST2 may play an important role in negatively regulating a putative protective role for IL-33 in the cardiovascular system. In humans, elevated circulating levels of sST2 are associated with adverse prognosis in patients with acute myocardial infarction (AMI) [11], [12] and heart failure [13], [14]. Recently, some studies have shown that sST2 levels are higher in patients with prevalent CVD who also have diabetes [12], [15], [16], [17]. However, so far the association between sST2 levels with cardiometabolic markers has not been carefully studied in any general population cohort.

We therefore sought to assess whether sST2 concentrations correlated with classic and novel markers of cardiovascular and diabetic risk in the cross-sectional psychological, social, and biological determinants of ill health (pSoBid) study, a representative sample drawn from a major UK city. Our hypothesis, based on protective effects of IL-33 with both vascular and metabolic outcomes was that sST2 would be related to both cardiovascular and diabetic risk factors and thus may be part of the common pathways underpinning cardiometabolic outcomes.

## Materials and Methods

### Study Population and Protocol

Study members were participants in the cross-sectional pSoBid study, established to test hypotheses directed at social determinants of ill health in particular pertaining to vascular disease. The design of the pSoBid study – including recruitment strategy, response rates, and study protocol has been described in detail elsewhere [18]. Briefly, 666 subjects (age 35–64 years) were recruited from 10 general practitioner practices within Glasgow, the largest city in Scotland; of whom 639 had the necessary data for the present analyses.

Participants were ranked on the basis of multiple deprivation indicators to define the least and most deprived areas in the NHS Greater Glasgow Health Board area, using criteria based on the Scottish Index for Multiple Deprivation (SIMD), which ranks small areas on the basis of multiple deprivation indicators across six domains, namely: income; employment; health; education, skills, and training; geographic access and telecommunications; and housing. Sampling was stratified to achieve an approximately equal distribution of the participants across males and females and age groups (35–44, 45–54 and 55–64 years) within the most (bottom 5% of SIMD score) and least deprived areas (top 20% of SIMD score). Summaries of participant characteristics are described in Tables S1.1, S1.2, S1.3. All subjects provided written consent. The study complied with the Declaration of Helsinki, and was approved by Glasgow Royal Infirmary Research Ethics Committee.

Participants underwent assessment of their medical history, undertook a physical examination (including measurement of blood pressure, body mass index (BMI), waist:hip ratio) and completed lifestyle questionnaires. Carotid ultrasound, to measure intima-media thickness and plaque presence, were carried out as previously described [18], [19]. A sample of fasted blood was collected and plasma was prepared, immediately frozen and stored at −80°C. Later blinded batched analysis of risk factor status included measurement of a range of classic (triglycerides, cholesterol) and novel CVD risk factors (C reactive protein [CRP], interleukin-6 [IL-6], soluble intercellular adhesion molecule 1 [sICAM-1], estimate glomerular filtration rate [eGFR], Fibrinogen) by methods as previously described [18], [19]. B-type natriuretic peptide [BNP] was measured on the Siemens ADVIA Centuar using a two-site sandwich Immunoassay. N-terminal [NT]-proBNP was measured on the Siemens Immulite using a two-site chemiluminescent immunometric assay. Plasma sST2 was quantified using a human IL-1 R4/ST2 DuoSet ELISA (R&D Systems), with a lower limit of detection of 3.9 pg/ml and mean intra-assay coefficient of variance (CV) of <5% and mean inter-assay CV of <12%.

### Statistical Analysis

Median sST2 levels (pg/ml) are presented graphically by gender and age category (35–44, 45–54, 55–64 years). We determined the associations between levels of sST2 in plasma and age, sex, and their interaction using linear regression models of logarithmically transformed sST2 as the outcome. We then assessed the associations between plasma sST2 and lifestyle factors (body mass index (BMI), physical activity, smoking, alcohol consumption and monthly aggregate intake of fruit and vegetables) and measures of socioeconomic status in adulthood (high or low Scottish Index of Multiple Deprivation (SIMD 2004) score, annual income and years of education) and in childhood (at 11 years of age: number of siblings, number of people per room as a child and leg length), using linear regression models adjusted for age, sex and their interaction. Results are presented as the relative change in sST2 for specified changes in lifestyle and socioeconomic factors, with 95% confidence intervals (CIs) and p-values.

Linear regression models (logistic regression for plaque presence) were then used to examine whether sST2 is a useful predictor of classic and novel markers of cardiovascular and diabetes risk, adjusted for age, sex and their interaction. Results of linear models are presented as either the relative or absolute change in the marker (depending on whether the marker was log-transformed prior to modelling or not) associated with a one standard deviation increase in log-transformed sST2, with a 95% CI and p-value. For plaque presence, the odds ratio associated with a one standard deviation increase in log-transformed sST2 is presented, with a 95% CI and p-value.

A sensitivity analysis of the results to subject medication use (either taking lipid lowering, anti-diabetic or anti-hypertensive drugs or not) was conducted. Where a significant interaction existed between sST2 and drug use as predictors, the drug use status specific estimates are presented.

The statistical software package R for Windows v2.14 was used for all analysis. No adjustments were made for multiple statistical comparisons, so our p-values must be considered as descriptive measures of the strength of evidence for the observed associations. Significance levels of <5% have been treated as suggestive of true associations, with smaller p-values representing greater levels of evidence.

## Results

### Plasma sST2 Levels Correlate with Age, Gender and Income

**Figure 1:**
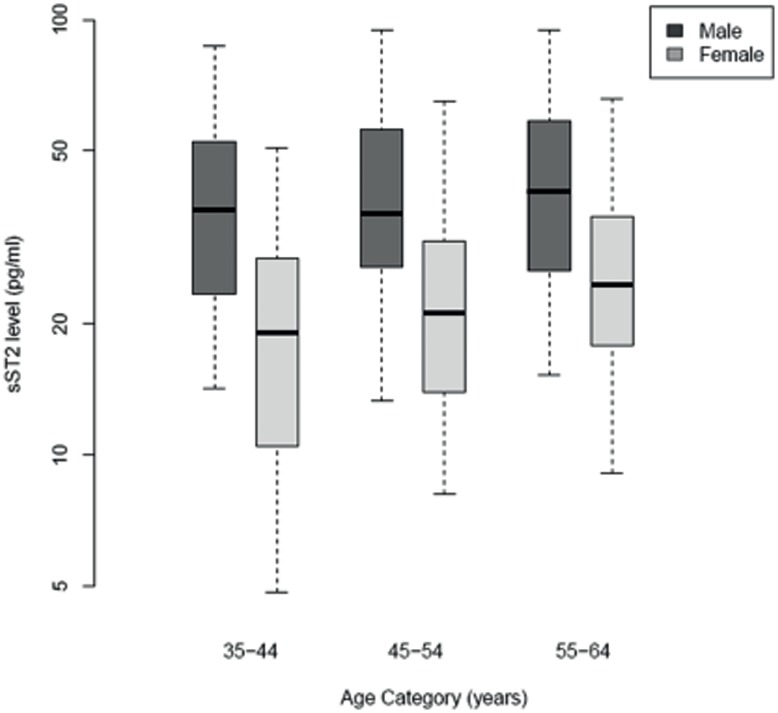
sST2 levels (pg/ml) by gender and age. Each box represents the median and upper/lower quartiles with the whiskers showing the 5^th^ and 95^th^ percentiles.

The median sST2 levels in plasma within specified age subgroups and split by gender are shown in Figure 1 and geometric mean, median and interquartile range values shown in Table S2. These data indicate overall higher sST2 concentrations in males than females, which is highly significant after adjusting for age (effect estimate 49% lower in women [95% CI 42% to 55%], p<0.0001) (Table 1). Levels of sST2 were associated with age in females only, with a relative increase of 24% (95% CI: 11.2% to 38.2%, p = 0.0001) for a 10 year increment in age, compared to a relative change in men of +3.9% (−7.0% to +16.3%), p = 0.50 (p-value for interaction, 0.027). In Table 1 we show the relation of sST2 with selected demographic, lifestyle, and socio-economic variables. Further regression analysis did not reveal any association of sST2 with markers of lifestyle such as body mass index (BMI), activity level, smoking, alcohol or fruit and vegetable intake. Analysis of markers of socioeconomic status (SES) demonstrated that levels of sST2 were 21.2% (6.2% –33.7%, p = 0.0073) lower in individuals with a salary >£45,000/annum in comparison to those with a salary <£15,000/annum independent of age and gender. Levels of sST2 were not associated with other markers of SES such as area deprivation, years of education or markers of childhood deprivation (number of siblings, number of people per room as a child and leg length).

**Table 1:** Associations (as relative effects) with 95% confidence intervals and p-values between sST2 as the outcome and selected demographic, lifestyle and socio-economic status predictor variables univariately and adjusted for age, sex and their interaction*.

Predictor Variable		Effect Estimate (95% CI), p-value	
		Univariate	Adjusted
**Demographic variables**			
Sex (Female vs. Male)	at age 50 years		0.51 (0.45, 0.58), p<0.0001
Age** (per 10 year increase)	Male Female		1.04 (0.93, 1.16), p = 0.5020 1.24(1.11, 1.38), p = 0.0001
**Lifestyle variables**			
BMI	(per 5 kg/m^2^)	1.04 (0.98, 1.11), p = 0.1736	1.05 (0.99, 1.11), p = 0.1246
Waist Circumference (per 5 cm increase)		1.05 (1.02, 1.07), p = 0.0004	1.01 (0.99, 1.04), p = 0.2686
Hip Circumference (per 5 cm increase)		1.00 (0.97, 1.03), p = 0.7670	1.00 (0.97, 1.03), p = 0.8512
Waist: Hip Ratio (per 10% increase)		1.30 (1.19, 1.41), p<0.0001	1.08 (0.98, 1.18), p = 0.1015
Activity Level (vs. Inactive)	Moderately InactiveModerately Active Active	0.81 (0.67, 0.99), p = 0.0421 0.82 (0.68, 0.98), p = 0.0292 0.87 (0.72, 1.05), p = 0.1443	0.90 (0.74, 1.08),p = 0.2555 0.92 (0.78, 1.10),p = 0.3583 0.90 (0.75, 1.07), p = 0.2331
Smoking (vs. Non Smokers)	Current Smokers	1.04 (0.88, 1.24), p = 0.6367	1.04 (0.89, 1.22), p = 0.5971
Diet Score	per score of 10	0.99 (0.98, 1.00), p = 0.1203	1.00 (0.99, 1.01), p = 0.7401
Alcohol Consumption (weekly units)	per 5 units	1.04 (1.02, 1.06), p = 0.0001	1.01 (0.99, 1.03), p = 0.1842
**Adult Markers of Social Deprivation**			
Deprivation (vs. Least Deprived)	Most Deprived	1.08 (0.94, 1.24), p = 0.2578	1.10 (0.97, 1.25), p = 0.1357
Annual Income (vs. <15,000)	16–25,000 26–35,00036–45,000 >45,000	0.97 (0.78, 1.21), p = 0.8174 0.92 (0.70, 1.19), p = 0.5097 0.96 (0.73, 1.26), p = 0.7694 0.84 (0.70, 1.01), p = 0.0643	0.96 (0.78, 1.17), p = 0.6801 0.96(0.75, 1.22), p = 0.7134 0.93 (0.72, 1.20),p = 0.5645 0.79 (0.66, 0.94), p = 0.0073
Education (vs. ≤11 yrs)	12–13 yrs 14–16 yrs≥17 yrs	1.00 (0.82, 1.23), p = 0.9992 1.03 (0.86, 1.23), p = 0.7675 0.97 (0.80, 1.17), p = 0.7111	1.08 (0.89, 1.31), p = 0.4270 1.06(0.90, 1.26), p = 0.4713 0.90 (0.75, 1.07),p = 0.2258
**Childhood Markers of Social Deprivation**			
Number of Siblings (vs. None)	1–2 3 ≥4	0.89 (0.72, 1.09), p = 0.2557 0.92 (0.72, 1.19), p = 0.5345 0.90 (0.70, 1.17), p = 0.4399	0.93 (0.76, 1.13), p = 0.4561 0.98(0.78, 1.23), p = 0.8604 0.98 (0.77, 1.24),p = 0.8628
People per Room (vs. ≤1)	>1, ≤1.5 >1.5	1.12 (0.95, 1.33), p = 0.1818 1.07 (0.91, 1.27), p = 0.4233	1.08 (0.92, 1.26), p = 0.3345 1.06(0.90, 1.24), p = 0.4765
Leg Length (vs. ≤75 cm)	75.1–80 cm 80.1–85 cm>85 cm	1.13 (0.91, 1.40), p = 0.2710 1.35 (1.09, 1.68), p = 0.0061 1.57 (1.25, 1.98), p = 0.0001	0.95 (0.77, 1.17), p = 0.6134 0.91(0.73, 1.15), p = 0.4290 0.93 (0.72, 1.20),p = 0.5977

* Associations after adjustment for age and sex with interaction. Regression models were fitted with log sST2 as the outcome. Effect estimates are the relative change in sST2 for a specified increase in continuous predictor variables, or compared to the stated reference group for categorical predictors.

** p-value = 0.0271 for the ineraction term of age and sex as predictors of log sST2.

### Plasma sST2 Levels Correlate with Markers of Diabetic, but not Cardiovascular, Risk

Associations between classic cardiovascular and diabetes risk factors and sST2 levels are reported in Table 2 and interquartile range values shown in Table S3. Individuals with higher sST2 levels had lower levels of HDL cholesterol, and increased levels of triglycerides, systolic blood pressure and waist:hip ratio. However, after adjustment for age, sex and their interaction, only the association with triglycerides (5% (0.6% to 9.6%) increase in triglycerides per one SD increase in log sST2, p = 0.025) was evident. There was no evidence of associations between sST2 and total or LDL cholesterol, or diastolic blood pressure.

**Table 2:** Associations between classical cardiovascular and metabolic risk factors (outcomes) and sST2 (predictor), univariately and adjusted for age and sex*.

Outcome	Effect Estimate (95% CI), p-value	
	Univariate	Adjusted
Total Cholesterol (mmol/l)	1.00 (0.98, 1.02) p = 0.8727	1.01 (0.99, 1.02) p = 0.4547
LDL-Cholesterol (mmol/l)	0.99 (0.97, 1.02) p = 0.6856	1.00 (0.97, 1.02) p = 0.7922
HDL-Cholesterol (mmol/l)	**0.96 (0.94, 0.98) p = 0.0003**	0.99 (0.97, 1.02) p = 0.5649
Triglycerides (mmol/l)	**1.08 (1.04, 1.13) p = 0.0001**	**1.05 (1.01, 1.10)** **p = 0.0250**
Systolic BP (mmHg)	**1.02 (1.01, 1.03) p = 0.0002**	1.01 (1.00, 1.02) p = 0.2683
Diastolic BP (mmHg)	1.01 (1.00, 1.02) p = 0.0642	1.00 (0.99, 1.02) p = 0.4205

* Regression models were fitted with classical risk factors as the outcome (all log transformed) and log sST2 as the predictor. Adjusted models include age, sex and their interaction.

Effect estimates are reported as the relative change in outcome associated with a one standard deviation in log sST2.

Associations of sST2 levels with emerging or novel cardiovascular and diabetes risk factors are shown in Table 3. Markers of insulin resistance and dysglycaemia (fasting glucose, insulin and the Homeostasis Model of Assessment [HOMA-IR]) all show signs of being associated with sST2, though only glucose was significant correlated following adjustment for age and sex (1.8% relative increase for one SD increase in log sST2; 0.3% to 3.2%, p = 0.019). Like other indices of obesity (BMI, waist:hip ratio) leptin was not related to sST2 levels following adjustment for age and sex (p = 0.066); the negative univariate association between leptin and sST2 was explained by an association between leptin and sex.

**Table 3:** Associations between novel cardiovascular and metabolic risk factors (outcomes) and sST2 (predictor), univariately and adjusted for age and sex*.

Outcome	Effect Estimate (95% CI), p-value	
	Univariate	Adjusted
Glucose (mmol/l)	**1.03 (1.01, 1.04) p = 0.0002**	**1.02 (1.00, 1.03) p = 0.0195**
Insulin (U/l)	1.05 (1.00, 1.11) p = 0.0521	1.04 (0.98, 1.10) p = 0.1940
HOMA-IR	**1.07 (1.01, 1.14) p = 0.0164**	1.05 (0.99, 1.12) p = 0.1246
Leptin (ng/ml)	**0.87 (0.81, 0.94) p = 0.0002**	1.06 (1.00, 1.13) p = 0.0660
ALT (U/l)	**1.12 (1.08, 1.16) p<0.0001**	**1.05 (1.01, 1.09) p = 0.0079**
GGT (U/l)	**1.21 (1.15, 1.28) p<0.0001**	**1.13 (1.07, 1.19) p<0.0001**
CRP (mg/l)	1.07 (0.98, 1.17) p = 0.1162	1.09 (1.00, 1.20) p = 0.0605
IL-6 (pg/ml)	**1.07 (1.02, 1.13) p = 0.0106**	1.05 (0.99, 1.11) p = 0.0824
sICAM-1 (pg/ml)	**1.05 (1.03, 1.07) p<0.0001**	**1.05 (1.02, 1.07) p = 0.0001**
Fibrinogen^(a)^ (g/l)	0.03 (−0.03, 0.08) p = 0.3111	0.05 (−0.01, 0.11) p = 0.1059
Cystatin C^(a)^ (mg/l)	**0.02 (0.01, 0.03) p = 0.0033**	0.01 (0, 0.02) p = 0.1494
eGFR^(a)^ (ml/min/1.73 m^2^)	0.88 (−0.28, 2.03) p = 0.1367	0.27 (−0.9, 1.45) p = 0.6509
BNP (pg/ml)	0.94 (0.85, 1.04) p = 0.2335	0.98 (0.88, 1.09) p = 0.7426
NT-proBNP (pg/ml)	1.03 (0.94, 1.13) p = 0.5874	1.07 (0.97, 1.18) p = 0.1584

* Regression models were fitted with novel risk factors as the outcome (all log transformed except for Fibrinogen, Cystatin C and eGFR) and log sST2 as the predictor. Adjusted models include age, sex and their interaction.

Effect estimates are reported as the relative change in outcome (except for (a), presented as the absolute change), associated with a one standard deviation increase in log sST2.

Despite being attenuated by age and sex adjustment, there were strong associations between sST2 and markers of hepatic function, with alanine aminotransaminase [ALT] and γ-glutamyl transferase [GGT] showing relative increases of 4.8% (1.3% to 8.6%, p = 0.0079) and 12.8% (6.9% to 19.1%, p<0.0001) for every SD increase in log sST2, respectively. Such associations remained robust to adjustment for alcohol intake (data not shown).

Of the markers of chronic inflammation (CRP, and IL-6) and endothelial dysfunction (sICAM-1), sST2 showed a significant association after age-sex adjustment only with sICAM-1, with a relative increase of 4.9% (2.5% to 7.3%, p = 0.0001) per SD increase in log sST2. There were no significant associations between sST2 and the other novel risk factors Fibrinogen, Cystatin C, eGFR, BNP, or NT-proBNP.

### sST2 and Atherosclerotic/Plaque Measures

Finally, we examined the association between sST2 levels and ultrasound measurements of atherosclerosis (common carotid intima-media thickness [c-IMT] and plaque presence; Table 4). We found a univariate association between sST2 and c-IMT (p = 0.0002), but no significant association following adjustment for age and sex. There was no evidence of an association between sST2 and plaque presence.

**Table 4:** Associations between measures of atherosclerotic burden (outcomes) and sST2 (predictor), univariately and adjusted for age and sex*.

Outcome	Effect Estimate (95% CI), p-value	
	Univariate	Adjusted
c-IMT (mm)	**1.03 (1.01, 1.05)** **p = 0.0002**	1.01 (0.99, 1.02) p = 0.4223
Plaque Presence^(b)^	1.18 (1.00, 1.40)p = 0.0539	1.01 (0.84, 1.21) p = 0.9195

* Regression models were fitted with log sST2 as the predictor. Adjusted models include age, sex and their interaction. For c-IMT, data were log transformed and a linear regression model was used. For plaque presence, a logistic regression model was used.

Effect estimates are presented as the relative change in c-IMT and the odds ratio for plaque presence associated with a one standard deviation increase in log sST2.

### Sensitivity Analysis

Tables S4, S5, S6 and S7 show the results of repeating the analysis adjusting for subject medication use. Subjects who were receiving lipid lowering, anti-diabetic or anti-hypertensive medications had 25% higher sST2 levels compared to those who were not. Associations between demographic and lifestyle variables and sST2 levels remained after adjusting for medication use. Associations between sST2 and ALT, GGT and sICAM-1 were similar to the main analysis, though sST2 was predictive of HDL-cholesterol and glucose for subjects who were on medications only.

## Discussion

The main finding of this study is that sST2, a decoy receptor for the recently described IL-1 family member IL-33, correlates with classic and novel markers of insulin resistance/endothelial dysfunction. Abnormal glucose metabolism defines diabetes and accounts for many of its symptoms and complications [20]. We show that sST2 significantly correlates with glucose levels in a general population. Our data extend a previous study showing higher sST2 levels in patients with type 2 diabetes and left ventricular dysfunction [16]. Furthermore, studies in patients with myocardial infarction or heart failure also showed that sST2 levels correlated with glucose [15], and were higher in patients with diabetes versus those who did not have diabetes [12], [15], [17]. Importantly, we also show correlation of sST2 with other markers linked to diabetes in addition to glucose. Circulating triglycerides, markers of ectopic fat in blood that are also linked to insulin resistance, correlated with sST2 levels, as did ALT and GGT, markers of liver function. Elevated levels of ALT and GGT are associated with T2D independent of other factors [21], [22], [23]. It is likely that these elevations in liver enzymes reflect increasing fatty liver and mechanisms linking excess hepatic fat with insulin resistance are now well established [24] such that fatty liver is an established risk factor for diabetes [25]. It is also important to recognize potential relationships between glycaemia and heart failure; sST2 is associated with prognosis of heart failure [12], [13], [14]. Poor glycaemic control is a known biomarker of heart failure severity and prognosis [26]. Furthermore, elevated GGT was also recently shown to be associated with increased risk of incident heart failure in men aged <70 years [27]. The present findings are therefore broadly consistent with a potential link between diabetes pathways, sST2, and heart failure. They suggest that the association of sST2 with heart failure prognosis may stem from its associations with metabolic perturbances.

The pSoBid study recruited subjects from least deprived and most deprived areas within Glasgow. Therefore, we were also able to investigate whether sST2 levels correlated with markers of socio-economic status. sST2 levels were lower in individuals with a higher salary, but did not correlate to other markers of social deprivation. Clearly, further studies are needed to confirm our observations but it is well known that socioeconomic factors are associated with higher diabetes risk.

We found increased levels of circulating sST2 in men, and increasing levels with age in women. These observations concur with previous studies that demonstrated a link between sST2 levels, age and male gender [15], [17]. A recent study investigated the mechanism behind this gender bias by measuring association of sST2 levels with androgen and oestrogen levels [28], but did not find an independent association of sST2 with sex hormones in healthy males and females. Therefore, the reason for the sex-specific difference of sST2 plasma concentrations remains uncertain, although it is now clear that men are at higher risk of diabetes than women at any given BMI [29].

This is the first study to suggest a link between circulating sICAM-1, a marker of endothelial dysfunction, and sST2 levels. A large body of evidence points towards the involvement of subclinical, low-grade inflammation and increased systemic levels of acute phase proteins, cytokines and adhesion molecules in patients with T2D [4]. Previous studies show that sICAM-1 predicts diabetes [30], and sICAM-1 associates more strongly with diabetic risk than cardiovascular risk [31]. Furthermore, it has been shown that endothelial cells can secrete sST2 in response to treatment with various inflammatory mediators [32]. Thus, the diabetic stressed vascular endothelium may be the source of the increased plasma levels of sST2 seen in diabetic patients. Other potential sources for sST2 secretion have been discussed by Mildner and co-workers and include lung epithelial cells and cardiac myocytes following activation with IL-1α/β and TNFα [33].

Interestingly, sST2 did not correlate with classic markers linked to CVD risk, including cholesterol, blood pressure, smoking, c-IMT or plaque presence. Many studies have shown that sST2 levels are increased and predict mortality in patients with AMI and/or heart failure [11], [12], [13], [14]. Thus it is possible that this increased mortality is not through an association with classic risk factors but rather via other pathways reflective of metabolic status. Of course, rather than a cause of vascular outcomes, sST2 could be a marker of worsening vascular or associated metabolic status.

The mechanistic link between circulating sST2 and diabetes is unclear at present but could be causal. The IL-33/ST2 pathway may participate in the inflammatory and remodeling processes of various tissues during diabetes. We have previously shown that IL-33 exerts protective effects on glucose metabolism and obesity in obese diabetic (*ob/ob*) mice [9]. Furthermore, IL-33 signaling through ST2L is proposed to limit destructive Th17 responses in a murine model of type 1 diabetes [34]. Increased levels of sST2 during diabetes may impair the protective effects of IL-33. It is thus possible that sST2 is not only a biomarker but may contribute to the pathogenesis of diabetes via IL-33 interactions.

There are some limitations to our study which merit consideration. These analyses were not pre-specified as part of the main pSoBid study protocol, so our findings should therefore be seen as exploratory, and in need of replication in other populations. Furthermore, we did not measure the ligand for sST2, IL-33. There is a lack of robust and sensitive assays for IL-33 meaning that this cytokine is difficult to detect in serum/plasma [15]. However, recent studies have highlighted the development of multiplex assays to measure low abundance IL-33 in serum or plasma and warrant further investigation in the context of diabetes and CVD [35]. Finally, our results stem from a cross-sectional study and thus we cannot infer predictive potential of sST2. Future prospective and genetic studies may be useful to address the predictive potential and causal role sST2 may have in cardiometabolic outcomes.

In summary, our findings suggest that sST2 levels associate more strongly with predictors of diabetes (triglycerides, glucose, ICAM-1, ALT, GGT), than with established cardiovascular risk predictors or surrogate vascular markers of atheroma (c-IMT or plaque). Further mechanistic studies determining how sST2 is linked to diabetes pathways, and how, in turn, these pathways may influence mortality risks in individuals with vascular conditions, would seem warranted and will likely offer new insights into the inflammatory paradigm for type 2 diabetes.

## Supporting Information

Table S1 **Summaries of study participant characteristics.** Table S1.1 shows the number of subjects and mean (sd) presented for continuous variables and the number (%) given for each level of categorical variable*. Table S1.2 shows classic cardiovascular and metabolic risk factors for each sex with the number of subjects and median (IQR)) presented*. Table S1.3 shows novel cardiovascular and metabolic risk factors for each sex. The number of subjects and geometric means (sd) are presented, except for ^(a)^, presented as the mean, and ^(b)^ as the number (%) with the presence of plaques*.
(DOCX)

Table S2 **Summaries of sST2 levels (pg/ml) by age and sex with the number of subjects, geometric mean (sd) and median (IQR) presented.**
(DOCX)

Table S3 **Summaries of selected variables by quartiles of sST2 (pg/ml). Median (IQR) are presented, except for Cystatin C where the mean is presented.**
(DOCX)

Table S4 Associations (as relative effects) with 95% confidence intervals and p-values between sST2 as the outcome and selected demographic, lifestyle and socio-economic status predictor variables univariately and adjusted for drug medication*, age, sex and their interaction**.
(DOCX)

Table S5 Associations between classical cardiovascular and metabolic risk factors (outcomes) and sST2 (predictor), univariately and adjusted for age, sex and medication use*. Where there is a different association between sST2 and the outcome for subjects taking medication and those not the individual associations are presented. Effect estimates are reported as the relative change in outcome associated with a one standard deviation in log sST2. P-value of interaction term in parenthesis.
(DOCX)

Table S6 Associations between novel cardiovascular and metabolic risk factors (outcomes) and sST2 (predictor), univariately and adjusted for age, sex and medication use*. Where there is a different association between sST2 and the outcome for subjects taking medication and those not the individual associations are presented. Effect estimates are reported as the relative change in outcome (except for (a), presented as the absolute change), associated with a one standard deviation increase in log sST2. P-value of interaction term in parenthesis.
(DOCX)

Table S7 Associations between measures of atherosclerotic burden (outcomes) and sST2 (predictor), univariately and adjusted for age, sex and medication use*. Effect estimates are presented as the relative change in c-IMT and the odds ratio for plaque presence associated with a one standard deviation increase in log sST2.
(DOCX)

## References

[1] Sarwar N, Gao P, Seshasai SR, Gobin R, Kaptoge S, et al (2010) Diabetes mellitus, fasting blood glucose concentration, and risk of vascular disease: a collaborative meta-analysis of 102 prospective studies. Lancet 375: 2215–2222. 20609967 10.1016/S0140-6736(10)60484-9 PMC2904878

[2] Batty GD, Shipley MJ, Marmot M, Smith GD (2004) Diabetes status and post-load plasma glucose concentration in relation to site-specific cancer mortality: findings from the original Whitehall study. Cancer Causes Control 15: 873–881. 15577289 10.1007/s10552-004-1050-z

[3] Sattar N, Wannamethee SG, Forouhi NG (2008) Novel biochemical risk factors for type 2 diabetes: pathogenic insights or prediction possibilities? Diabetologia 51: 926–940. 18392804 10.1007/s00125-008-0954-7

[4] Kolb H, Mandrup-Poulsen T (2005) An immune origin of type 2 diabetes? Diabetologia 48: 1038–1050. 15864529 10.1007/s00125-005-1764-9

[5] Fernandez-Real JM, Pickup JC (2012) Innate immunity, insulin resistance and type 2 diabetes. Diabetologia 55: 273–278. 22124608 10.1007/s00125-011-2387-y

[6] Tominaga S (1989) A putative protein of a growth specific cDNA from BALB/c-3T3 cells is highly similar to the extracellular portion of mouse interleukin 1 receptor. FEBS Lett 258: 301–304. 2532153 10.1016/0014-5793(89)81679-5

[7] Schmitz J, Owyang A, Oldham E, Song Y, Murphy E, et al (2005) IL-33, an interleukin-1-like cytokine that signals via the IL-1 receptor-related protein ST2 and induces T helper type 2-associated cytokines. Immunity 23: 479–490. 16286016 10.1016/j.immuni.2005.09.015

[8] Miller AM, Xu D, Asquith DL, Denby L, Li Y, et al (2008) IL-33 reduces the development of atherosclerosis. J Exp Med 205: 339–346. 18268038 10.1084/jem.20071868 PMC2271006

[9] Miller AM, Asquith DL, Hueber AJ, Anderson LA, Holmes WM, et al (2010) Interleukin-33 induces protective effects in adipose tissue inflammation during obesity in mice. Circ Res 107: 650–658. 20634488 10.1161/CIRCRESAHA.110.218867 PMC4254700

[10] Sanada S, Hakuno D, Higgins LJ, Schreiter ER, McKenzie AN, et al (2007) IL-33 and ST2 comprise a critical biomechanically induced and cardioprotective signaling system. J Clin Invest 117: 1538–1549. 17492053 10.1172/JCI30634 PMC1865027

[11] Shimpo M, Morrow DA, Weinberg EO, Sabatine MS, Murphy SA, et al (2004) Serum levels of the interleukin-1 receptor family member ST2 predict mortality and clinical outcome in acute myocardial infarction. Circulation 109: 2186–2190. 15117853 10.1161/01.CIR.0000127958.21003.5A

[12] Sabatine MS, Morrow DA, Higgins LJ, MacGillivray C, Guo W, et al (2008) Complementary roles for biomarkers of biomechanical strain ST2 and N-terminal prohormone B-type natriuretic peptide in patients with ST-elevation myocardial infarction. Circulation 117: 1936–1944. 18378613 10.1161/CIRCULATIONAHA.107.728022 PMC4273564

[13] Weinberg EO, Shimpo M, Hurwitz S, Tominaga S, Rouleau JL, et al (2003) Identification of serum soluble ST2 receptor as a novel heart failure biomarker. Circulation 107: 721–726. 12578875 10.1161/01.cir.0000047274.66749.fe

[14] Januzzi JL Jr, Peacock WF, Maisel AS, Chae CU, Jesse RL, et al (2007) Measurement of the interleukin family member ST2 in patients with acute dyspnea: results from the PRIDE (Pro-Brain Natriuretic Peptide Investigation of Dyspnea in the Emergency Department) study. J Am Coll Cardiol 50: 607–613. 17692745 10.1016/j.jacc.2007.05.014

[15] Dhillon OS, Narayan HK, Quinn PA, Squire IB, Davies JE, et al (2011) Interleukin 33 and ST2 in non-ST-elevation myocardial infarction: comparison with Global Registry of Acute Coronary Events Risk Scoring and NT-proBNP. Am Heart J 161: 1163–1170. 21641364 10.1016/j.ahj.2011.03.025

[16] Fousteris E, Melidonis A, Panoutsopoulos G, Tzirogiannis K, Foussas S, et al (2011) Toll/interleukin-1 receptor member ST2 exhibits higher soluble levels in type 2 diabetes, especially when accompanied with left ventricular diastolic dysfunction. Cardiovasc Diabetol 10: 101. 22104207 10.1186/1475-2840-10-101 PMC3229462

[17] Kohli P, Bonaca MP, Kakkar R, Kudinova AY, Scirica BM, et al (2012) Role of ST2 in Non-ST-Elevation Acute Coronary Syndrome in the MERLIN-TIMI 36 Trial. Clin Chem 58: 257–266. 22096031 10.1373/clinchem.2011.173369 PMC4277435

[18] Velupillai YN, Packard CJ, Batty GD, Bezlyak V, Burns H, et al (2008) Psychological, social and biological determinants of ill health (pSoBid): study protocol of a population-based study. BMC Public Health 8: 126. 18426568 10.1186/1471-2458-8-126 PMC2386810

[19] Deans KA, Bezlyak V, Ford I, Batty GD, Burns H, et al (2009) Differences in atherosclerosis according to area level socioeconomic deprivation: cross sectional, population based study. Bmj 339: b4170. 19861369 10.1136/bmj.b4170 PMC2768777

[20] Seshasai SR, Kaptoge S, Thompson A, Di Angelantonio E, Gao P, et al (2011) Diabetes mellitus, fasting glucose, and risk of cause-specific death. N Engl J Med 364: 829–841. 21366474 10.1056/NEJMoa1008862 PMC4109980

[21] Sattar N, Scherbakova O, Ford I, O’Reilly DS, Stanley A, et al (2004) Elevated alanine aminotransferase predicts new-onset type 2 diabetes independently of classical risk factors, metabolic syndrome, and C-reactive protein in the west of Scotland coronary prevention study. Diabetes 53: 2855–2860. 15504965 10.2337/diabetes.53.11.2855

[22] Wannamethee SG, Shaper AG, Lennon L, Whincup PH (2005) Hepatic enzymes, the metabolic syndrome, and the risk of type 2 diabetes in older men. Diabetes Care 28: 2913–2918. 16306554 10.2337/diacare.28.12.2913

[23] Sattar N, McConnachie A, Ford I, Gaw A, Cleland SJ, et al (2007) Serial metabolic measurements and conversion to type 2 diabetes in the west of Scotland coronary prevention study: specific elevations in alanine aminotransferase and triglycerides suggest hepatic fat accumulation as a potential contributing factor. Diabetes 56: 984–991. 17395744 10.2337/db06-1256

[24] Samuel VT, Liu ZX, Qu X, Elder BD, Bilz S, et al (2004) Mechanism of hepatic insulin resistance in non-alcoholic fatty liver disease. J Biol Chem 279: 32345–32353. 15166226 10.1074/jbc.M313478200

[25] Ghouri N, Preiss D, Sattar N (2010) Liver enzymes, nonalcoholic fatty liver disease, and incident cardiovascular disease: a narrative review and clinical perspective of prospective data. Hepatology 52: 1156–1161. 20658466 10.1002/hep.23789

[26] Suskin N, McKelvie RS, Burns RJ, Latini R, Pericak D, et al (2000) Glucose and insulin abnormalities relate to functional capacity in patients with congestive heart failure. Eur Heart J 21: 1368–1375. 10952826 10.1053/euhj.1999.2043

[27] Wannamethee SG, Whincup PH, Shaper AG, Lennon L, Sattar N (2012) gamma-Glutamyltransferase, Hepatic Enzymes, and Risk of Incident Heart Failure in Older Men. Arterioscler Thromb Vasc Biol. 10.1161/ATVBAHA.111.240457 22223732

[28] Dieplinger B, Egger M, Poelz W, Gabriel C, Haltmayer M, et al (2011) Soluble ST2 is not independently associated with androgen and estrogen status in healthy males and females. Clin Chem Lab Med 49: 1515–1518. 21663467 10.1515/CCLM.2011.239

[29] Logue J, Walker JJ, Colhoun HM, Leese GP, Lindsay RS, et al (2011) Do men develop type 2 diabetes at lower body mass indices than women? Diabetologia 54: 3003–3006. 21959958 10.1007/s00125-011-2313-3 PMC4220585

[30] Song Y, Manson JE, Tinker L, Rifai N, Cook NR, et al (2007) Circulating levels of endothelial adhesion molecules and risk of diabetes in an ethnically diverse cohort of women. Diabetes 56: 1898–1904. 17389327 10.2337/db07-0250 PMC1952236

[31] Sattar N, Murray HM, Welsh P, Blauw GJ, Buckley BM, et al (2009) Are elevated circulating intercellular adhesion molecule 1 levels more strongly predictive of diabetes than vascular risk? Outcome of a prospective study in the elderly. Diabetologia 52: 235–239. 19030842 10.1007/s00125-008-1217-3

[32] Bartunek J, Delrue L, Van Durme F, Muller O, Casselman F, et al (2008) Nonmyocardial production of ST2 protein in human hypertrophy and failure is related to diastolic load. J Am Coll Cardiol 52: 2166–2174. 19095135 10.1016/j.jacc.2008.09.027 PMC2637465

[33] Mildner M, Storka A, Lichtenauer M, Mlitz V, Ghannadan M, et al (2010) Primary sources and immunological prerequisites for sST2 secretion in humans. Cardiovasc Res 87: 769–777. 20363761 10.1093/cvr/cvq104

[34] Zdravkovic N, Shahin A, Arsenijevic N, Lukic ML, Mensah-Brown EP (2009) Regulatory T cells and ST2 signaling control diabetes induction with multiple low doses of streptozotocin. Mol Immunol 47: 28–36. 19356801 10.1016/j.molimm.2008.12.023

[35] Kuhn E, Addona T, Keshishian H, Burgess M, Mani DR, et al (2009) Developing multiplexed assays for troponin I and interleukin-33 in plasma by peptide immunoaffinity enrichment and targeted mass spectrometry. Clin Chem 55: 1108–1117. 19372185 10.1373/clinchem.2009.123935 PMC2865473

