# Supplementary material for: Soluble ST2 Associates with Diabetes but Not Established Cardiovascular Risk Factors: A New Inflammatory Pathway of Relevance to Diabetes?
Source: PLoS One. 2012 Oct 24;7(10):e47830. doi: 10.1371/journal.pone.0047830 (PMC3480428; doi:10.1371/journal.pone.0047830)
Supplement: Table S1 — Summaries of study participant characteristics. Table S1.1 shows the number of subjects and mean (sd) presented for continuous variables and the number (%) given for each level of categorical variable*. Table S1.2 shows classic cardiovascular and metabolic risk factors for each sex with the number of subjects and median (IQR)) presented*. Table S1.3 shows novel cardiovascular and metabolic risk factors for each sex. The number of subjects and geometric means (sd) are presented, except for (a), presented as the mean, and (b) as the number (%) with the presence of plaques*. (DOCX) [file pone.0047830.s001.docx]

**Table S1: Summaries of study participant characteristics.**

| **Table S1.1: Summaries of all variables for each sex with the number of subjects and mean (sd) presented for continuous variables and the number (%) given for each level of categorical variable*** | | | | |
| --- | --- | --- | --- | --- |
|  |  | All Subjects | Male | Female |
| Age (years) |  | 639 (0) 51.79 (8.22) | 316 (0) 51.82 (8.12) | 323 (0) 51.77 (8.34) |
| BMI |  | 635 (4) 27.80 (5.54) | 315 (1) 27.60 (4.53) | 320 (3) 28.00 (6.37) |
| Waist Circumference |  | 637 (2) 94.67 (14.13) | 316 (0) 98.34 (12.30) | 321 (2) 91.06 (14.89) |
| Hip Circumference |  | 633 (6) 105.46 (11.53) | 315 (1) 104.56 (8.39) | 318 (5) 106.35 (13.92) |
| Waist:Hip Ratio |  | 633 (6) 0.90 (0.08) | 315 (1) 0.94 (0.07) | 318 (5) 0.85 (0.07) |
| Activity Level | Inactive Moderately Inactive Moderately Active Active | 224 (35.1%) 116 (18.2%) 156 (24.4%) 143 (22.4%) | 122 (38.6%) 50 (15.8%) 65 (20.6%) 79 (25.0%) | 102 (31.6%) 66 (20.4%) 91 (28.2%) 64 (19.8%) |
| Smoking Status | Non-smoker Smoker | 464 (76.6%) 142 (23.4%) | 220 (76.7%) 67 (23.3%) | 244 (76.5%) 75 (23.5%) |
| Diet Score |  | 639 (0) 78.31 (54.44) | 316 (0) 70.13 (51.25) | 323 (0) 86.32 (56.32) |
| Weekly Alcohol Consumption (units) |  | 639 (0) 11.08 (17.25) | 316 (0) 16.54 (22.19) | 323 (0) 5.73 (7.06) |
| Deprivation Group | Affluent Deprived | 332 (52.0%) 307 (48.0%) | 168 (53.2%) 148 (46.8%) | 164 (50.8%) 159 (49.2%) |
| Income | < 15,000 16-25,000 26-35,000 36-45,000 > 45,000 | 185 (31.0%) 102 (17.1%) 61 (10.2%) 55 ( 9.2%) 193 (32.4%) | 81 (27.1%) 48 (16.1%) 25 ( 8.4%) 30 (10.0%) 115 (38.5%) | 104 (35.0%) 54 (18.2%) 36 (12.1%) 25 ( 8.4%) 78 (26.3%) |
| Years of Education | ≤ 11 yrs 12-13 yrs 14-16 yrs ≥ 17 yrs | 218 (34.1%) 112 (17.5%) 164 (25.7%) 145 (22.7%) | 98 (31.0%) 46 (14.6%) 80 (25.3%) 92 (29.1%) | 120 (37.2%) 66 (20.4%) 84 (26.0%) 53 (16.4%) |
| Number of Siblings | None 1-2 3 ≥ 4 | 90 (14.1%) 333 (52.3%) 112 (17.6%) 102 (16.0%) | 47 (14.9%) 168 (53.2%) 56 (17.7%) 45 (14.2%) | 43 (13.4%) 165 (51.4%) 56 (17.4%) 57 (17.8%) |
| Number of People per Room | ≤ 1 > 1, ≤ 1.5 > 1.5 | 236 (37.0%) 196 (30.8%) 205 (32.2%) | 115 (36.4%) 106 (33.5%) 95 (30.1%) | 121 (37.7%) 90 (28.0%) 110 (34.3%) |
| * The numbers in parentheses after the number of observations represent the numbers of missing values | | | | |

| **Table S1.2: Summaries of classic cardiovascular and metabolic risk factors for each sex with the number of subjects and median (IQR)) presented*** | | | | |
| --- | --- | --- | --- | --- |
|  |  | All Subjects | Male | Female |
| Total Cholesterol (mmol/l) |  | 639 (0) 5.05 (4.43, 5.80) | 316 (0) 5.00 (4.29, 5.80) | 323 (0) 5.10 (4.55, 5.85) |
| LDL-Cholesterol (mmol/l) |  | 635 (4) 3.00 (2.40, 3.60) | 313 (3) 3.00 (2.35, 3.60) | 322 (1) 3.00 (2.45, 3.55) |
| HDL-Cholesterol (mmol/l) |  | 639 (0) 1.30 (1.10, 1.60) | 316 (0) 1.20 (1.00, 1.40) | 323 (0) 1.50 (1.25, 1.70) |
| Triglycerides (mmol/l) |  | 639 (0) 1.25 (0.92, 1.75) | 316 (0) 1.33 (1.00, 1.96) | 323 (0) 1.15 (0.88, 1.50) |
| Diastolic BP (mmHg) |  | 636 (3) 81.0 (74.0, 88.0) | 313 (3) 82.0 (75.0, 90.0) | 323 (0) 81.0 (73.0, 87.0) |
| Systolic BP (mmHg) |  | 636 (3) 135.0 (124.0, 147.0) | 313 (3) 138.0 (127.0, 149.0) | 323 (0) 130.0 (118.5, 144.0) |
| * The numbers in parentheses after the number of observations represent the numbers of missing values | | | | |

| **Table S1.3: Summaries of novel cardiovascular and metabolic risk factors for each sex. The number of subjects and geometric means (sd) are presented, except for ^(a)^, presented as the mean, and ^(b)^ as the number (%) with the presence of plaques*** | | | | |
| --- | --- | --- | --- | --- |
|  |  | All Subjects | Male | Female |
| Glucose (mmol/l) |  | 608 (31) 5.10 (4.80, 5.40) | 298 (18) 5.20 (4.90, 5.50) | 310 (13) 5.00 (4.70, 5.30) |
| Insulin (U/l) |  | 605 (34) 5.63 (3.64, 8.55) | 296 (20) 5.85 (3.95, 9.67) | 309 (14) 5.23 (3.53, 8.01) |
| HOMA-IR |  | 591 (48) 1.26 (0.78, 2.01) | 288 (28) 1.37 (0.88, 2.25) | 303 (20) 1.13 (0.76, 1.88) |
| Leptin (ng/ml) |  | 631 (8) 13.50 (7.10, 27.90) | 314 (2) 7.80 (4.93, 12.90) | 317 (6) 24.70 (13.70, 41.00) |
| ALT (U/l) |  | 636 (3) 22.0 (17.0, 31.0) | 314 (2) 27.0 (22.0, 37.0) | 322 (1) 18.5 (15.0, 24.0) |
| GGT (U/l) |  | 636 (3) 26.0 (19.0, 41.0) | 314 (2) 32.0 (23.0, 52.8) | 322 (1) 22.0 (16.0, 31.0) |
| CRP (mg/l) |  | 630 (9) 1.60 (0.68, 3.41) | 311 (5) 1.59 (0.66, 3.14) | 319 (4) 1.61 (0.70, 3.67) |
| IL-6 (pg/ml) |  | 627 (12) 1.63 (1.01, 2.75) | 310 (6) 1.64 (0.98, 2.86) | 317 (6) 1.62 (1.09, 2.63) |
| ICAM-1 (pg/ml) |  | 634 (5) 251.4 (217.5, 312.4) | 312 (4) 253.2 (218.4, 320.4) | 322 (1) 248.8 (217.1, 310.7) |
| Fibrinogen^(a)^ (g/l) |  | 627 (12) 3.36 (0.71) | 308 (8) 3.29 (0.69) | 319 (4) 3.43 (0.73) |
| Cystatin C^(a)^ (mg/l) |  | 636 (3) 0.96 (0.14) | 314 (2) 0.97 (0.12) | 322 (1) 0.96 (0.16) |
| eGFR^(a)^ (ml/min/1.73m^2^) |  | 636 (3) 86.47 (14.84) | 314 (2) 89.71 (14.33) | 322 (1) 83.30 (14.66) |
| BNP (pg/ml) |  | 481 (158) 13.27 (7.39, 24.47) | 198 (118) 11.14 (6.27, 21.95) | 283 (40) 14.51 (8.55, 25.76) |
| NT-proBNP (pg/ml) |  | 485 (154) 36.6 (10.0, 73.2) | 199 (117) 22.7 (10.0, 67.1) | 286 (37) 44.2 (21.8, 76.4) |
| c-IMT (mm) |  | 592 (47) 0.67 (0.59, 0.76) | 292 (24) 0.69 (0.60, 0.78) | 300 (23) 0.66 (0.59, 0.73) |
| Plaque Presence ^(b)^ | Yes | 319 (51.0%) | 176 (56.8%) | 143 (45.3%) |
| * The numbers in parentheses after the number of observations represent the numbers of missing values | | | | |
