# Supplementary material for: Soluble ST2 Associates with Diabetes but Not Established Cardiovascular Risk Factors: A New Inflammatory Pathway of Relevance to Diabetes?
Source: PLoS One. 2012 Oct 24;7(10):e47830. doi: 10.1371/journal.pone.0047830 (PMC3480428; doi:10.1371/journal.pone.0047830)
Supplement: Table S2 — Summaries of sST2 levels (pg/ml) by age and sex with the number of subjects, geometric mean (sd) and median (IQR) presented. (DOCX) [file pone.0047830.s002.docx]

| **Table S2: Summaries of sST2 levels (pg/ml) by age and sex with the number of subjects, geometric mean (sd) and median (IQR) presented.** | | | |
| --- | --- | --- | --- |
|  | All Subjects | Male | Female |
| Age 35-44 | 160  22.6 (2.7) 23.9 (14.9, 40.7) | 72 35.3 (1.9) 36.6 (23.5, 52.4) | 88  15.6 (3.0) 19.1 (10.5, 28.4) |
| Age 45-54 | 223 27.8 (2.4) 29.9 (19.2, 43.8) | 120 37.6 (1.9) 36.0 (27.1, 55.9) | 103  19.5 (2.7) 21.3 (14.0, 31.0) |
| Age 55-64 | 256  29.9 (2.3) 30.8 (20.7, 47.6) | 124  38.9 (1.7) 40.2 (26.6, 58.5) | 132  23.4 (2.5) 24.7 (17.9, 35.2) |
