# Supplementary material for: Soluble ST2 Associates with Diabetes but Not Established Cardiovascular Risk Factors: A New Inflammatory Pathway of Relevance to Diabetes?
Source: PLoS One. 2012 Oct 24;7(10):e47830. doi: 10.1371/journal.pone.0047830 (PMC3480428; doi:10.1371/journal.pone.0047830)
Supplement: Table S3 — Summaries of selected variables by quartiles of sST2 (pg/ml). Median (IQR) are presented, except for Cystatin C where the mean is presented. (DOCX) [file pone.0047830.s003.docx]

| **Table S3: Summaries of selected variables by quartiles of sST2 (pg/ml). Median (IQR) are presented, except for Cystatin C where the mean is presented.** | | | | | |
| --- | --- | --- | --- | --- | --- |
| Variable | All Subjects | sST2 Quartiles (pg/ml) | | | |
|  |  | < 18.6 | 18.6 - 29.16 | 29.161 - 46.655 | > 46.655 |
| HDL-Cholesterol (mmol/l) | 639 (0) 1.30 (1.10, 1.60) | 160 (0) 1.45 (1.20, 1.65) | 160 (0) 1.30 (1.15, 1.65) | 159 (0) 1.25 (1.05, 1.55) | 160 (0) 1.30 (1.05, 1.55) |
| Triglycerides (mmol/l) | 639 (0) 1.25 (0.92, 1.75) | 160 (0) 1.10 (0.90, 1.45) | 160 (0) 1.15 (0.90, 1.75) | 159 (0) 1.40 (1.00, 1.92) | 160 (0) 1.27 (0.95, 2.00) |
| Systolic BP (mmHg) | 636 (3) 135.0 (124.0, 147.0) | 160 (0) 130.0 (116.8, 144.0) | 160 (0) 134.0 (122.0, 144.2) | 158 (1) 135.0 (125.0, 147.8) | 158 (2) 138.0 (128.0, 151.0) |
| Diastolic BP (mmHg) | 636 (3) 81.0 (74.0, 88.0) | 160 (0) 80.0 (73.8, 87.0) | 160 (0) 81.0 (75.0, 87.2) | 158 (1) 81.0 (73.0, 89.0) | 158 (2) 83.0 (76.0, 91.0) |
| Glucose (mmol/l) | 608 (31) 5.10 (4.80, 5.40) | 156 (4) 5.00 (4.80, 5.30) | 153 (7) 5.00 (4.70, 5.40) | 152 (7) 5.20 (4.80, 5.50) | 147 (13) 5.10 (4.80, 5.55) |
| Insulin (U/l) | 605 (34) 5.63 (3.64, 8.55) | 158 (2) 5.43 (3.50, 7.94) | 151 (9) 5.14 (3.34, 8.63) | 148 (11) 5.79 (4.11, 8.34) | 148 (12) 6.20 (3.71, 10.35) |
| HOMA-IR | 591 (48) 1.26 (0.78, 2.01) | 155 (5) 1.19 (0.75, 1.84) | 149 (11) 1.10 (0.71, 2.01) | 144 (15) 1.29 (0.90, 2.00) | 143 (17) 1.43 (0.83, 2.47) |
| Leptin (ng/ml) | 631 (8) 13.5 (7.1, 27.9) | 159 (1) 16.0 (9.4, 33.1) | 158 (2) 16.9 (7.7, 28.9) | 158 (1) 10.4 (6.3, 25.8) | 156 (4) 10.9 (6.2, 22.6) |
| ALT (U/l) | 636 (3) 22.0 (17.0, 31.0) | 159 (1) 19.0 (14.0, 25.0) | 159 (1) 22.0 (17.0, 28.0) | 159 (0) 24.0 (19.0, 32.0) | 159 (1) 25.0 (20.0, 36.5) |
| GGT (U/l) | 636 (3) 26.0 (19.0, 41.0) | 159 (1) 21.0 (15.0, 32.0) | 159 (1) 25.0 (18.0, 37.0) | 159 (0) 28.0 (21.0, 40.5) | 159 (1) 33.0 (23.0, 61.0) |
| IL-6 (pg/ml) | 627 (12) 1.63 (1.01, 2.75) | 158 (2) 1.51 (0.98, 2.54) | 157 (3) 1.52 (0.98, 2.61) | 156 (3) 1.69 (1.04, 2.61) | 156 (4) 1.88 (1.14, 3.36) |
| ICAM-1 (pg/ml) | 634 (5) 251.4 (217.5, 312.4) | 159 (1) 234.6 (210.5, 278.1) | 159 (1) 245.7 (215.4, 301.0) | 158 (1) 251.2 (224.8, 324.8) | 158 (2) 278.5 (233.1, 340.3) |
| Cystatin C^(a)^ (mg/l) | 636 (3) 0.94 (0.88, 1.03) | 159 (1) 0.93 (0.87, 1.01) | 159 (1) 0.94 (0.87, 1.02) | 159 (0) 0.94 (0.87, 1.04) | 159 (1) 0.96 (0.90, 1.05) |
| c-IMT (mm) | 592 (47) 0.67 (0.59, 0.76) | 152 (8) 0.65 (0.58, 0.73) | 143 (17) 0.67 (0.60, 0.74) | 151 (8) 0.69 (0.60, 0.77) | 146 (14) 0.69 (0.60, 0.78) |
