# Supplementary material for: Soluble ST2 Associates with Diabetes but Not Established Cardiovascular Risk Factors: A New Inflammatory Pathway of Relevance to Diabetes?
Source: PLoS One. 2012 Oct 24;7(10):e47830. doi: 10.1371/journal.pone.0047830 (PMC3480428; doi:10.1371/journal.pone.0047830)
Supplement: Table S4 — Associations (as relative effects) with 95% confidence intervals and p-values between sST2 as the outcome and selected demographic, lifestyle and socio-economic status predictor variables univariately and adjusted for drug medication*, age, sex and their interaction**. (DOCX) [file pone.0047830.s004.docx]

| **Table S4: Associations (as relative effects) with 95% confidence intervals and p-values between sST2 as the outcome and selected demographic, lifestyle and socio-economic status predictor variables univariately and adjusted for drug medication*, age, sex and their interaction**** | | | |
| --- | --- | --- | --- |
| Predictor Variable |  | Effect Estimate (95% CI), p-value | |
|  |  | Univariate | Adjusted |
| **Demographic variables** | | | |
| Sex (Female vs. Male) | at age 50 years |  | 0.51 (0.45, 0.59), p<0.0001 |
| Age***  (per 10 year increase) | Male Female |  | 0.99 (0.88, 1.11), p=0.8609  1.19 (1.06, 1.33), p=0.0026 |
| **Lifestyle variables** | | | |
| BMI | (per 5 kg/m^2^) | 1.04 (0.98, 1.11), p=0.1736 | 1.03 (0.97, 1.09), p=0.3837 |
| Waist Circumference (per 5cm increase) |  | 1.05 (1.02, 1.07), p=0.0004 | 1.01 (0.98, 1.03), p=0.6588 |
| Hip Circumference (per 5cm increase) |  | 1.00 (0.97, 1.03), p=0.7670 | 1.00 (0.97, 1.02), p=0.7741 |
| Waist: Hip Ratio  (per 10% increase) |  | 1.30 (1.19, 1.41), p<0.0001 | 1.06 (0.96, 1.16), p=0.2552 |
| Activity Level (vs. Inactive) | Moderately Inactive Moderately Active Active | 0.81 (0.67, 0.99), p=0.0421 0.82 (0.68, 0.98), p=0.0292 0.87 (0.72, 1.05), p=0.1443 | 0.91 (0.75, 1.09), p=0.3064 0.94 (0.79, 1.12), p=0.4803 0.92 (0.77, 1.10), p=0.3440 |
| Smoking (vs. Non Smokers) | Current Smokers | 1.04 (0.88, 1.24), p=0.6367 | 1.02 (0.87, 1.20), p=0.7805 |
| Diet Score | per score of 10 | 0.99 (0.98, 1.00), p=0.1203 | 1.00 (0.99, 1.01), p=0.8936 |
| Alcohol Consumption (weekly units) | per 5 units | 1.04 (1.02, 1.06), p=0.0001 | 1.01 (1.00, 1.03), p=0.1417 |
| Current Medications* | Yes | 1.36 (1.16, 1.60), p=0.0001 | 1.25 (1.06, 1.46), p=0.0073 |
| **Adult Markers of Social Deprivation** | | | |
| Deprivation (vs. Least Deprived) | Most Deprived | 1.08 (0.94, 1.24), p=0.2578 | 1.06 (0.93, 1.21), p=0.3495 |
| Annual Income (vs. < 15,000) | 16-25,000 26-35,000 36-45,000 > 45,000 | 0.97 (0.78, 1.21), p=0.8174 0.92 (0.70, 1.19), p=0.5097 0.96 (0.73, 1.26), p=0.7694 0.84 (0.70, 1.01), p=0.0643 | 0.97 (0.80, 1.19), p=0.7900 0.98 (0.77, 1.24), p=0.8393 0.97 (0.76, 1.26), p=0.8416 0.82 (0.69, 0.98), p=0.0310 |
| Education (vs. ≤ 11 yrs) | 12-13 yrs 14-16 yrs ≥ 17 yrs | 1.00 (0.82, 1.23), p=0.9992 1.03 (0.86, 1.23), p=0.7675 0.97 (0.80, 1.17), p=0.7111 | 1.09 (0.90, 1.31), p=0.3863 1.09 (0.92, 1.30), p=0.2982 0.93 (0.78, 1.12), p=0.4449 |
| **Childhood Markers of Social Deprivation** | | | |
| Number of Siblings (vs. None) | 1-2 3 ≥ 4 | 0.89 (0.72, 1.09), p=0.2557 0.92 (0.72, 1.19), p=0.5345 0.90 (0.70, 1.17), p=0.4399 | 0.93 (0.77, 1.13), p=0.4780 0.98 (0.78, 1.24), p=0.8761 0.97 (0.77, 1.23), p=0.7988 |
| People per Room (vs. ≤ 1) | > 1, ≤ 1.5 > 1.5 | 1.12 (0.95, 1.33), p=0.1818 1.07 (0.91, 1.27), p=0.4233 | 1.08 (0.92, 1.26), p=0.3451 1.03 (0.88, 1.21), p=0.6838 |
| Leg Length (vs. ≤ 75cm) | 75.1-80cm 80.1-85cm > 85cm | 1.13 (0.91, 1.40), p=0.2710 1.35 (1.09, 1.68), p=0.0061 1.57 (1.25, 1.98), p=0.0001 | 0.95 (0.77, 1.17), p=0.6308 0.94 (0.75, 1.18), p=0.5813 0.96 (0.75, 1.24), p=0.7813 |
| *Medication indicates if the subject is receiving lipid lowering, anti-hypertension or anti-diabetes medications. **Associations after adjustment for medication use, age and sex with interaction. Regression models were fitted with log sST2 as the outcome. Effect estimates are the relative change in sST2 for a specified increase in continuous predictor variables, or compared to the stated reference group for categorical predictors. ***p-value = 0.0212 for the interaction term of age and sex as predictors of log sST2. | | | |
