# Supplementary material for: Soluble ST2 Associates with Diabetes but Not Established Cardiovascular Risk Factors: A New Inflammatory Pathway of Relevance to Diabetes?
Source: PLoS One. 2012 Oct 24;7(10):e47830. doi: 10.1371/journal.pone.0047830 (PMC3480428; doi:10.1371/journal.pone.0047830)
Supplement: Table S5 — Associations between classical cardiovascular and metabolic risk factors (outcomes) and sST2 (predictor), univariately and adjusted for age, sex and medication use*. Where there is a different association between sST2 and the outcome for subjects taking medication and those not the individual associations are presented. Effect estimates are reported as the relative change in outcome associated with a one standard deviation in log sST2. P-value of interaction term in parenthesis. (DOCX) [file pone.0047830.s005.docx]

| **Table S5: Associations between classical cardiovascular and metabolic risk factors (outcomes) and sST2 (predictor), univariately and adjusted for age, sex and medication use*. Where there is a different association between sST2 and the outcome for subjects taking medication and those not the individual associations are presented. Effect estimates are reported as the relative change in outcome associated with a one standard deviation in log sST2. P-value of interaction term in parenthesis.** | | | |
| --- | --- | --- | --- |
| Outcome |  | Effect Estimate (95% CI), p-value | |
|  |  | Univariate | Adjusted |
| Total Cholesterol (mmol/l) |  | 1.00 (0.98, 1.02), p=0.8727 | 1.01 (1.00, 1.03), p=0.1152 |
| LDL-Cholesterol (mmol/l) |  | 0.99 (0.97, 1.02), p=0.6856 | 1.01 (0.98, 1.03), p=0.5884 |
| HDL-Cholesterol (mmol/l) |  | **0.96 (0.94, 0.98), p=0.0003** | 0.99 (0.97, 1.01), p=0.4210 ^a^  **1.06 (1.01, 1.12), p=0.0251** ^b^  **(p=0.0158)** |
| Triglycerides (mmol/l) |  | **1.08 (1.04, 1.13), p=0.0001** | 1.04 (1.00, 1.08), p=0.0778 |
| Systolic BP (mmHg) |  | **1.02 (1.01, 1.03), p=0.0002** | 1.00 (0.99, 1.02), p=0.4376 |
| Diastolic BP (mmHg) |  | 1.01 (1.00, 1.02), p=0.0642 | 1.00 (0.99, 1.01), p=0.5689 |
| * Regression models were fitted with classical risk factors as the outcome (all log transformed) and log sST2 as the predictor. Adjusted models include current use lipid lowering, anti-hypertension or anti-diabetes medications, age, sex and their interaction. a: Not on medication b: On medication | | | |
