# Supplementary material for: Soluble ST2 Associates with Diabetes but Not Established Cardiovascular Risk Factors: A New Inflammatory Pathway of Relevance to Diabetes?
Source: PLoS One. 2012 Oct 24;7(10):e47830. doi: 10.1371/journal.pone.0047830 (PMC3480428; doi:10.1371/journal.pone.0047830)
Supplement: Table S6 — Associations between novel cardiovascular and metabolic risk factors (outcomes) and sST2 (predictor), univariately and adjusted for age, sex and medication use*. Where there is a different association between sST2 and the outcome for subjects taking medication and those not the individual associations are presented. Effect estimates are reported as the relative change in outcome (except for (a), presented as the absolute change), associated with a one standard deviation increase in log sST2. P-value of interaction term in parenthesis. (DOCX) [file pone.0047830.s006.docx]

| **Table S6: Associations between novel cardiovascular and metabolic risk factors (outcomes) and sST2 (predictor), univariately and adjusted for age, sex and medication use*. Where there is a different association between sST2 and the outcome for subjects taking medication and those not the individual associations are presented. Effect estimates are reported as the relative change in outcome (except for (a), presented as the absolute change), associated with a one standard deviation increase in log sST2. P-value of interaction term in parenthesis.** | | | |
| --- | --- | --- | --- |
| Outcome |  | Effect Estimate (95% CI), p-value | |
|  |  | Univariate | Adjusted |
| Glucose (mmol/l) |  | **1.03 (1.01, 1.04), p=0.0002** | 1.01 (0.99, 1.02), p=0.3327 ^a^  **1.05 (1.01, 1.09), p=0.0073** ^b^  **(p=0.0325)** |
| Insulin  (U/l) |  | 1.05 (1.00, 1.11), p=0.0521 | 1.03 (0.97, 1.09), p=0.3417 |
| HOMA-IR |  | **1.07 (1.01, 1.14), p=0.0164** | 1.04 (0.98, 1.10), p=0.2375 |
| Leptin (ng/ml) |  | **0.87 (0.81, 0.94), p=0.0002** | 1.04 (0.98, 1.11), p=0.2012 |
| ALT  (U/l) |  | **1.12 (1.08, 1.16), p<0.0001** | **1.05 (1.01, 1.08), p=0.0111** |
| GGT  (U/l) |  | **1.21 (1.15, 1.28), p<0.0001** | **1.11 (1.06, 1.18), p=0.0001** |
| CRP (mg/l) |  | 1.07 (0.98, 1.17), p=0.1162 | 1.07 (0.97, 1.17), p=0.1700 |
| IL-6 (pg/ml) |  | **1.07 (1.02, 1.13), p=0.0106** | 1.04 (0.98, 1.10), p=0.1930 |
| sICAM-1 (pg/ml) |  | **1.05 (1.03, 1.07), p<0.0001** | **1.04 (1.02, 1.07), p=0.0004** |
| Fibrinogen^(a)^ (g/l) |  | 0.03 (-0.03, 0.08), p=0.3111 | 0.04 (-0.02, 0.1), p=0.1983 |
| Cystatin C^(a)^ (mg/l) |  | **0.02 (0.01, 0.03), p=0.0033** | 0.01 (-0.01, 0.02), p=0.2924 |
| eGFR^(a)^ (ml/min/1.73m^2^) |  | 0.88 (-0.28, 2.03), p=0.1367 | 0.34 (-0.84, 1.52), p=0.5746 |
| BNP  (pg/ml) |  | 0.94 (0.85, 1.04), p=0.2335 | 0.96 (0.87, 1.07), p=0.4543 |
| NT-proBNP  (pg/ml) |  | 1.03 (0.94, 1.13), p=0.5874 | 1.05 (0.95, 1.15), p=0.3487 |
| * Regression models were fitted with novel risk factors as the outcome (all log transformed except for Fibrinogen, Cystatin C and eGFR) and log sST2 as the predictor. Adjusted models include current use lipid lowering, anti-hypertension or anti-diabetes medications, age, sex and their interaction. a: Not on medication b: On medication | | | |
