# Supplementary material for: Soluble ST2 Associates with Diabetes but Not Established Cardiovascular Risk Factors: A New Inflammatory Pathway of Relevance to Diabetes?
Source: PLoS One. 2012 Oct 24;7(10):e47830. doi: 10.1371/journal.pone.0047830 (PMC3480428; doi:10.1371/journal.pone.0047830)
Supplement: Table S7 — Associations between measures of atherosclerotic burden (outcomes) and sST2 (predictor), univariately and adjusted for age, sex and medication use*. Effect estimates are presented as the relative change in c-IMT and the odds ratio for plaque presence associated with a one standard deviation increase in log sST2. (DOCX) [file pone.0047830.s007.docx]

| **Table S7: Associations between measures of atherosclerotic burden (outcomes) and sST2 (predictor), univariately and adjusted for age, sex and medication use*. Effect estimates are presented as the relative change in c-IMT and the odds ratio for plaque presence associated with a one standard deviation increase in log sST2.** | | | |
| --- | --- | --- | --- |
| Outcome |  | Effect Estimate (95% CI), p-value | |
|  |  | Univariate | Adjusted |
| c-IMT (mm) |  | **1.03 (1.01, 1.05), p=0.0002** | 1.00 (0.99, 1.02), p=0.5618 |
| Plaque Presence^(b)^ |  | 1.18 (1.00, 1.40), p=0.0539 | 0.99 (0.82, 1.19), p=0.9152 |
| * Regression models were fitted with log sST2 as the predictor. Adjusted models include current use lipid lowering, anti-hypertension or anti-diabetes medications,age, sex and their interaction. For c-IMT, data were log transformed and a linear regression model was used. For plaque presence, a logistic regression model was used. | | | |
